# Platelet aggregation and risk of stent thrombosis or bleeding in interventionally treated diabetic patients with acute coronary syndrome

**DOI:** 10.1186/s12872-016-0433-x

**Published:** 2016-12-08

**Authors:** K. Kukula, M. Klopotowski, P. K. Kunicki, J. Jamiolkowski, A. Debski, P. Bekta, M. Polanska-Skrzypczyk, Z. Chmielak, A. Witkowski

**Affiliations:** 1 Department of Interventional Cardiology and Angiology, Institute of Cardiology, Alpejska 42, 04-628 Warsaw, Poland; 2 Clinical Pharmacology Unit, Department of Clinical Biochemistry, Institute of Cardiology, Warsaw, Poland; 3 Department of Public Health, Medical University of Bialystok, Bialystok, Poland

**Keywords:** Platelet aggregation, Diabetes, Acute coronary syndrome, Coronary intervention, Stent thrombosis

## Abstract

**Background:**

Platelet aggregation monitoring in diabetic patients treated with coronary interventions (PCI) for an acute coronary syndrome (ACS) is a promising way of optimizing treatment and outcomes in this high risk group. The aim of the study was to verify whether clopidogrel response measured by Multiplate analyzer (ADPtest) in diabetic ACS patients treated with PCI predicts the risk of stent thrombosis or cardiovascular mortality and bleeding.

**Methods:**

Into this prospective, observational study 206 elective PCI patients were enrolled. Two cutoff points of ADPtest were used in analysis to divide patients into groups. One (345 AU x min) was calculated based on ROC curve analysis; this cutoff provided the best ROC curve fit, although it did not reach statistical significance. The other (468 AU x min) was accepted based on the consensus of the Working Group on On-Treatment Platelet Reactivity. The risk of stent thrombosis and mortality was assessed using Cox regression analysis and Kaplan-Meier curves.

**Results:**

The risk of stent thrombosis was higher in the group of patients with impaired clopidogrel response for either cutoff value (for >354 AU x min - HR 12.33; 95% CI 2.49–61.1; *P* = 0.002). Cardiovascular mortality was also higher in the impaired clopidogrel response group (for >354 AU x min - HR 10.58; 95% CI 2.05–54.58; *P* = 0.005). We did not find a clear relation of increased clopidogrel response to the risk of bleeding.

**Conclusions:**

The results of this study show that in diabetic ACS patient group treated with PCI an impaired platelet response to clopidogrel measured by the Multiplate analyzer results in increased risk of stent thrombosis and cardiac death.

## Background

Acute coronary syndromes (ACS) in diabetic patients are associated with increased overall risk compared to the general ACS population despite unquestionable advances in treating these patients [1]. Most patients are treated with percuatenous intervention (PCI) with stent implantation [2] while a minority are referred for coronary artery bypass grafting (CABG). Still, recurrent ischemia is a common clinical phenomenon among patients with diabetes [3]. For some time now newer antiplatelet agents, such as ticagrelor and prasugrel, have been preferentially advocated for use in ACS instead of clopidogrel. However, in many centers worldwide the routinely used antiplatelet agent in most settings is still clopidogrel [4, 5]. It is generally recognized that many patients exhibit diminished response to clopidogrel [6]. This problem may be and probably is even more important in diabetic ACS patients than in the unselected patient population treated with PCI [7, 8]. The clinical relevance of inadequate reduction of platelet aggregation by antiplatelet agents has now been a matter of debate for some years [9–15]. Many observational studies show it to be of paramount importance, while some randomized trials are much less conclusive [16–19].

There are several methods of assessing platelet aggregation accepted for clinical use, such as Verify Now (Accriva Diagnostics, San Diego, CA, USA), Multiplate (Roche Diagnostics, Rotkreuz, Switzerland), VASP (Diagnostica Stago, Asnieres sur Seine, France) and some others, less standardized [20–22]. Based on available evidence from randomized trials none of these methods is currently recommended for routine clinical use as the efficacy of routine platelet function tests for guiding the treatment has not been confirmed [23, 24].

Most studies examining platelet aggregation, either ADP or aspirin induced, were done in cohorts of stable coronary artery disease (CAD) patients after elective PCI procedures or in unselected all-comer populations [25, 26]. There are less studies in patients with ACS and the data on diabetic patients is mostly derived from subgroup analyses of large all-comer studies [27–30].

Hence, the aim of the present study was to prospectively assess whether the degree of platelet aggregation inhibition in a high risk population of diabetic ACS patients on clopidogrel, treated with PCI, affects outcome. The clinical outcome endpoints were certain or probable stent thrombosis, and bleeding. The study also attempted to elucidate what is the best cutoff point of aggregations values for predicting stent thrombosis and bleeding in this population.

## Methods

This was a prospective single-center observational study into which 206 diabetic patients admitted for ACS to the Institute of Cardiology in Warsaw were enrolled in the years 2011–2014.

Ethics, consent and permissions: The study protocol was approved by the local Ethical Review Board and conducted in accordance with the Declaration of Helsinki. The study was financed by a National Science Centre grant NN 402381438.

Consecutive diabetic patients admitted for ACS treated by successful PCI with stent implantation were included. ACS, either STEMI or NSTEMI in case of the analyzed group, was defined according to the Third Universal Definition of Myocardial Infarction [31]. Patients on chronic anticoagulants or medications known to influence platelet aggregation (such as non-steroid anti-inflammatory agents or steroids) were excluded.

The primary endpoint of the study was definite or probable stent thrombosis as defined by the Academic Research Consortium criteria [32] and major or moderate bleeding as assessed by the GUSTO [33] study criteria.

The secondary endpoints of the study were cardiovascular and overall mortality [34] within one year.

All patients were loaded with 600 mg of clopidogrel either prior to or immediately after admission and received 300 mg of aspirin. Angiography and PCI procedures were performed according to the guidelines of the European Society of Cardiology [23], with no restriction as to stent selection, which was left to the discretion of the operator. Post-dilatation with non-compliant balloons for procedure optimization was strongly encouraged.

### Platelet function assessment

ADP-induced platelet reactivity assessment was performed directly prior to patient discharge, but no earlier than in the fourth day after admission. The MEA (multiple electrode platelet aggregometry) analyzer Multiplate (Dynabyte, Munich, Germany) was used. The method has been reapeatedly described previously [35, 36]. In short, 3 ml of whole blood is collected into a tube containing a direct thrombin inhibitor. After dilution with saline and agitation while incubating at 37 °C for 3 min in test cuvettes, 6.4 μmol ADP was added. Platelet aggregation was recorded continuously for 6 min and plotted against time in AU x min arbitrary units representing the area under the curve (ADPtest).

The same analyzer was also used to assess platelet response to acetylsalicylic acid (ASA). In this case 0.5 mM solution of arachidonic acid was used as agonist (ASPItest).

Study personnel was blinded to the results of the tests – the test result database was accessed for analysis only after completing patient follow-up.

### Clinical follow-up

Clinical follow-up was done by outpatient visit or telephone interview with the patient at 30 days and 12 months after the PCI procedure. Data was entered into a computer database. In case of endpoint or adverse event occurrence every effort was made to obtain and review source documentation.

### Statistics

Group (sample) size was calculated based on data from literature such as the risk of ischemic events and percentage of patients on clopidogrel showing reduced response to the drug. The Power and Sample Size Calculator software was used. We inferred that combined endpoint frequency will be 20% in patients with inadequate clopidogrel response and 8% in patients with adequate response. In order to achieve 80% power (beta 0.8) at the significance level of 0.05 we needed to include 192 patients. Data was collected on preprinted forms and entered into a database. Statistical analysis was performed using SPSS 22 (IBM Corp., USA) and SAS 9.2 (SAS Institute, Cary, NC, USA) software. Normally distributed variables were presented as means and standard deviation, while non-normally distribute variables as medians and interquartile range. Categorical variables were presented as percentages of patients exhibiting a given trait. Depending on distribution variables were analyzed using Student’s *t*-test or Mann-Whitney *U*-test. Chi-square test was used for categorical variables. If the number of values was less than 5, Fisher’s exact test was used. Correlation between linear variables was assessed with Pearson’s test. The analysis of endpoints was performed using the Kaplan-Meier method (K-M). The censored observation model was employed. The curves were compared using the log rank test. In order to elucidate variables influencing outcome a Cox proportional hazard model was used with the forward conditional Wald variant. A ROC (receiver operator characteristic) curve analysis was calculated to determine optimal platelet aggregation cutoff values both for thrombotic episodes and for bleeding. A P value of less than 0.05 was considered statistically significant in all analyses.

## Results

A total of 206 diabetic patients treated with PCI for ACS were enrolled in the trial, out of which 6 (2.9%) did not complete follow-up.

### The ADP-induced platelet reactivity and the risk of definite or probable stent thrombosis

During one year follow-up there were 7 cases of definite stent thrombosis and 1 case of sudden cardiac death adjudicated as probable stent thrombosis. They occurred between day 3 and day 360 after the PCI procedure.

The patients were divided into two groups — adequate response to clopidogrel and impaired response to clopidogrel based on the ADP-induced platelet aggregation MEA Multiplate test result. Two cutoff values of ADP-dependent platelet aggregation as assessed by MEA were analyzed.

The first cutoff point equaling 468 AU x min was accepted based on previous literature data and the consensus of the Working Group on On-Treatment Platelet Reactivity. This cutoff point had been elucidated after analyzing lower risk patient groups (mostly non-diabetic patients undergoing elective procedures) than in the case of this paper. This however is the generally accepted reference cutoff for the Multiplate test.

**Fig. 1:**
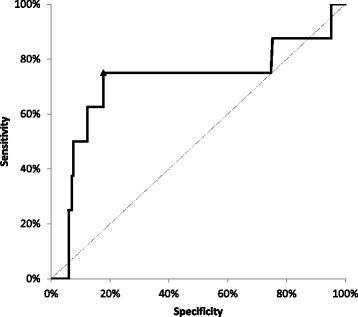
ROC curve calculated for ADP-dependent platelet aggregation as assessed by MEA (ADPtest)

The second, calculated based on ROC curve analysis, was 345 AU x min. Despite this being the best fit, the statistical significance was not reached, possibly due to low endpoint frequency (ROC curve analysis: sensitivity 75%, specificity 82%, AUC under the ROC curve 0.72, 95% confidence interval 0.47–0.97, *P* = 0.08; Fig. 1). However, this cutoff related better to study endpoints in the analyzed cohort.

The clinical and demographic characteristics of the studied group are presented in Table 1.

**Table 1:** Clinical and demographic data of the studied group with respect to platelet aggregation assessed by MEA Multiplate analyzer at the cutoff point of 345 AUmin

	ADPtest	ADPtest	*P*-value
	<345 AU × min	345 ≥ AU × min	
	*n* = 160	*n* = 40	
Age (years) (mean ± SD)	66.5 ± 11.1	69.6 ± 10.8	*p* = 0.084
Male (%)	109 (68%)	29 (72.5%)	*p* = 0.593
STEMI (%)	74 (53%)	20 (50%)	*p* = 0.695
ASPItest (AU x min)	88.9 ± 73.0	173.1 ± 150.0	*p* < 0.001
Drug eluting stent	132 (94%)	33 (83%)	*p* = 0.911
Stent diameter (mm) (median)	3.0	3.0	*p* = 0.450
Stent (s) length (mm)	18.0	22.0	*p* = 0.247
Inflation pressure (atm) (median)	16.0	16.0	*p* = 0.809
Fibrinogen (mg/dL) (mean ± SD)	451 ± 133	480 ± 138	*p* = 0.358
LDL (mmol/L) (mean ± SD)	2.83 ± 1.1	2.58 ± 0.9	*p* = 0.278
Creatinine (umol/L) (mean ± SD)	92.9 ± 34.4	103.4 ± 67.6	*p* = 0.421
hsCRP (mg/dL) (median)	0.4	0.5	*p* = 0.396
Platelet count (10^3^/ml) (mean ± SD)	204 ± 60	223 ± 70	*p* = 0.117
White blood count (10^3^/ml) (mean ± SD)	8.65	8.45	*p* = 0.793
Hemoglobin (mg/dL) (median)	13.8	12.8	*p* = 0.067
Insulin therapy	46 (33%)	15 (38%)	*P* = 0.293

The degree of inhibition of arachidonic acid induced platelet aggregation (ASPItest) was the only difference between the two groups of patients — above (impaired response) and below (adequate response) the 468 AU x min cutoff point. The ASPI test result difference remained statistically significant also when the standard 345 AU x min cutoff point was used.

### Primary endpoint analysis at the ADPtest cutoff point of 468 AUmin

**Fig. 2:**
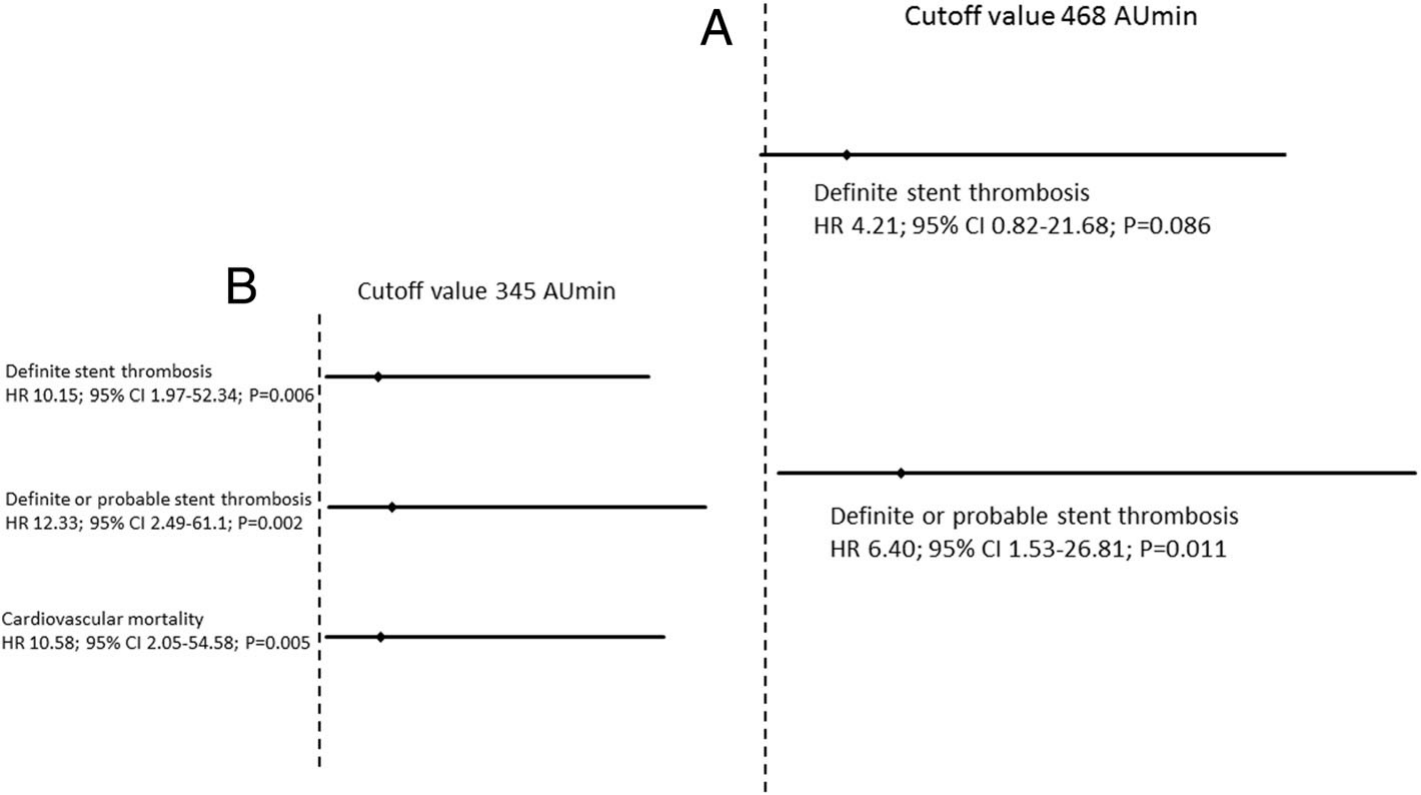
**a** Hazard ratio values of stent thrombosis (as described in the Figure) plotted as HR with 95% confidence intervals for standard MEA cutoff value of 468 AUmin; Inset (**b**) Hazard ratio values of stent thrombosis and cardiovascular mortality (as described in the Figure) for the cutoff of 345 AUmin that allowed for better risk stratification in the studied cohort

In the case of the advocated reference platelet aggregation cutoff point of 468 AUmin, the risk of definite stent thrombosis was not significantly higher in the impaired clopidogrel response group (HR 4.21; 95% CI 0.82–21.68; *P* = 0.086) compared to the remaining group. However, the risk of definite or probable stent thrombosis was significantly higher in the impaired response group (HR 6.40; 95% CI 1.53–26.81; *P* = 0.011; Fig. 2a).

### Primary endpoint analysis at the ADPtest cutoff point of 345 AUmin

When dividing patients into two groups at the cutoff point of 345 AU x min (based on ROC curve analysis), the risk of definite stent thrombosis proved to be significantly higher in the impaired clopidogrel response group (above 345 AU x min; HR 10.15; 95% CI 1.97–52.34; *P* = 0.006). Similarly, the risk of definite or probable stent thrombosis was significantly higher in the impaired response group (HR 12.33; 95% CI 2.49–61.1; *P* = 0.002; Fig. 2b).

### Risk of stent thrombosis and other potential influencing factors

In the Cox model analysis there was no increased risk of stent thrombosis related to the response to aspirin assessed by MEA, despite this parameter being different between groups irrespectively of the cutoff point (compare Table 1). The only other factor related to the risk of definite or probable stent thrombosis was hsCRP serum concentration (HR 1.11; 95% CI 1.01–1.22; *P* = 0.03).

None of the other clinical or demographic factors out of those presented in Table 1 were related to the risk of stent thrombosis in the Cox model.

### The analysis of secondary endpoints

Cardiovascular death within one year occurred in 7 patients. The risk of cardiovascular death was significantly higher for patients with ADPtest values above 345 AU x min compared to the remaining group (Cox regression, HR 10.58; 95% CI 2.05–54.58; *P* = 0.005), but not in case of the cutoff point of 468 AU x min (*P* = 0.076). Furthermore, the risk of cardiovascular mortality was also higher when the ADPtest was used as a linear variable (HR 1.002; 95% CI 1.0001–1.005; *P* = 0.04).

Overall, 11 patients died during the one-year follow-up. The risk of any-cause death was significantly higher only in case of patients with ADPtest values above 345 AU x min (HR 3.48; 95% CI 1.06–11.39; *P* = 0.04) compared to the remaining group.

### Bleeding

Major bleeding as defined by GUSTO II criteria occurred in 5 patients, while major or moderate bleeding in 24 patients. Based on ROC curve analysis for ADP-induced platelet aggregation and major or moderate bleeding, the best cutoff point discriminating the population at risk for bleeding was calculated. The MEA aggregation value of 278 AUmin proved the best cutoff point for bleeding in this population (sensitivity 88%, specificity 42%; ROC curve AUC 0.61, 95% CI 0.51–0.72; *P* = 0.034). However, in the Cox proportional hazards model neither the cutoff point of 278 AU x min nor the cutoff point used for unselected groups in earlier literature (188 AU x min) was significantly related to increased bleeding risk.

## Discussion

In this prospective observational study we analyzed the risk of stent thrombosis and bleeding in diabetic patients presenting with an acute coronary syndrome and pretreated with clopidogrel and aspirin before PCI. The relative risk was assessed in relation to the extent of ADP-induced platelet aggregation measured by MEA Multiplate analyzer.

We found that increased platelet reactivity (or inadequate platelet inhibition) in patients on clopidogrel is related to increased risk of stent thrombosis and cardiovascular mortality within one year. In the studied cohort, the optimal discriminating cutoff point of platelet reactivity above which the risk of stent thrombosis is higher was found to be 345 AU x min. The optimal cutoff point for the risk of bleeding was, in turn, 278 AU x min.

To date, the clinical implications of platelet reactivity in patients after PCI were assessed in numerous studies using a number of different methods, also the MEA Multiplate analyzer [37–39]. Most of the studied patient cohorts consisted of elective low stent thrombosis risk patients or unselected all-comer PCI patients [40–42]. These and other prior studies suggest that the risk of stent thrombosis is highest in acute coronary syndrome patients, while diabetes is an additional important concomitant condition increasing this risk [43–50].

Observational studies in elective patients, consecutive patient cohorts and the several ACS patient groups, with few exceptions, quite uniformly showed an increased risk of stent thrombosis in patients with inadequate inhibition of platelet aggregation [51–56]. Different methods of laboratory assessment of platelet aggregation were used with the MEA Multiplate method repeatedly showing consistent results across several studies [20, 35, 57].

This evidence spurred randomized trials that have to date failed to confirm the relationship of platelet aggregation values assessed by laboratory methods and stent thrombosis. The results of several such trials have been published. One of them was a subgroup study within the TRILOGY-ACS trial [58], where conservatively treated patients had platelet reactivity assessed with the VerifyNow assay. No difference was found between patients treated with clopidogrel or more aggressively – with prasugrel. In other studies, such as the GRAVITAS, ARCTIC and TRIGGER-PCI [16, 59–62], platelet reactivity was assessed using different methods in patients after PCI procedures, on different treatment regimens, but the extent of platelet inhibition did not translate into a difference in the risk of stent thrombosis or ischemic events. Two trials, a non-randomized MADONNA study [63] and the small randomized study by Hazarbasanova et al. [29] showed that modifying therapy based on platelet aggregation assessment using the Multiplate analyzer might bear on outcome.

The present study was undertaken as we felt there was lack of direct specific data concerning platelet reactivity and outcome in high risk populations and because of conflicting evidence of the above-mentioned studies.

Despite enrolling a comparatively small cohort and relatively few occurrences of primary endpoint we have shown that increased platelet reactivity in clopidogrel treated diabetic ACS patients after PCI is related to increased risk of stent thrombosis. The optimal cutoff point, above which platelet reactivity may be considered overt and related to stent thrombosis in the studied cohort was 345 AU x min, less than the generally accepted cutoff elucidated in earlier studies. However, also the standard cutoff of 468 AU x min allowed to discriminate the group of patients at increased risk for stent thrombosis. It must be noted that this lower cutoff, although providing better risk stratification did not reach significance in ROC curve analysis. The difference between the optimal cutoff between this and earlier studies must therefore be considered an approximation and is most probably the effect of different cohorts studied. In most trials to date the majority of enrolled patients were low risk patients. In this study of diabetic patients presenting with ACS the extent of platelet inhibition in order to avoid stent thrombosis may be expected to be higher [comp. 30]. The study also showed that patients with higher ADP-induced platelet reactivity on clopidogrel also show diminished response to aspirin [comp. 26]. We have not, however, seen an increase in the risk of stent thrombosis in relation to arachidonic acid induced platelet reactivity alone.

Decreased platelet aggregation invariably results in an increase of bleeding risk as has been repeatedly reported in previous studies [35, 64, 65]. In the analyzed cohort one may reasonably expect both increased stent thrombosis and increased bleeding risk compared to unselected populations. This was confirmed by finding that the optimal cutoff point for increased bleeding in the studied group was 278 AU x min – higher than the “standard” cutoff for bleeding of 188 AU x min elucidated in previous studies. However, we did not find a clear relation of this cutoff to the risk of bleeding.

Based on the data acquired in the study one may think that the therapeutic window for clopidogrel assessed with the MEA Multiplate analyzer is more narrow for our study group than for an unselected lower risk cohort of patients after PCI. Newer antiplatelet drugs, such as prasugrel and ticagrelor act more uniformly than clopidogrel and provide greater inhibition of platelet aggregation. Therefore, as clinical data from other studies show [41, 44], in a high risk population such as the one analyzed, they provide better protection against stent thrombosis at the cost of greater bleeding risk. Platelet function monitoring using, for example, the MEA Multiplate method in high risk cohorts, could lead to a reduction in bleeding without unduly increasing the risk of stent thrombosis. Such monitored therapy need not be confined just to one antiplatelet drug and should allow for modifying therapy if necessary [66]. This approach requires confirmation in randomized trials, especially since there may be several seemingly attractive strategies of monitored antiplatelet therapy.

### Study limitations

The major inherent limitation of this study is its observational character, not allowing for causal analysis. The study cohort is relatively small. The primary study endpoint occurred in only 8 patients which precluded meaningful multifactorial analysis. On the other hand, apart from ADP-induced platelet aggregation, practically no other analyzed factors correlated with increased risk of stent thrombosis upon single factor analysis, so it may be considered an independent association. Platelet reactivity was measured only once in every patient, but at a period where drug effect should be full. However, the absence of repeated measurements would fail to identify patients who stopped taking the drug despite recommendations. Patients in this study were treated with clopidogrel, which at the time of study commencement was the only routinely used antiplatelet drug in our country. At present, newer antiplatelet agents are preferentially recommended that inhibit platelet aggregation more profoundly, but their use is still not dominant in most areas. It is difficult to predict how they would influence study outcomes as far as stent thrombosis and bleeding are concerned.

## Conclusions

This work shows that in a high risk population of patients with ACS and diabetes treated with stent implantation and receiving clopidogrel, higher on-treatment platelet reactivity is related to increased risk of stent thrombosis at 1-year follow-up. Whether platelet reactivity-guided therapy may decrease the risk of stent thrombosis and patient prognosis must be resolved in randomized trials, a few of which are already underway.

## Abbreviations

ACS: Acute coronary syndrome
ADP: Adenosine-diphosphate
ADPtest: Multiplate analyzer test for assessing platelet aggregation on clopidogrel
ASA: Acetylsalicylic acid
ASPItest: Multiplate analyzer test for assessing platelet aggregation on aspirin
AU x min: Arbitrary units used for expressing aggregation values measured with the Multiplate analyzer
10 AU x min is equivalent to 1 U: Another arbitrary unit also used for the same purpose
CABG: Coronary artery bypass grafting
CAD: Coronary artery disease
ECG: Electrocardiogram
GUSTO: Global utilization of streptokinase and TPA for occluded arteries trial
hsCRP: High sensitivity C-reactive protein
MEA: Multiple electrode platelet aggregometry
NSTEMI: Non-ST elevation myocardial infarction
PCI: Percutaneous coronary intervention
ROC: Receiver operator characteristic
STEMI: ST elevation myocardial infarction

## References

[1] Roffi M, Patrono C, Collet JP (2016). 2015 ESC guidelines for the management of acute coronary syndromes in patients presenting without persistent ST-segment elevation: task force for the management of acute coronary syndromes in patients presenting without persistent ST-segment elevation of the European society of cardiology (ESC). Eur Heart J.

[2] Levine GN, Bates ER, Blankenship JC (2011). 2011 ACCF/AHA/SCAI guideline for percutaneous coronary intervention: a report of the American college of cardiology foundation/American heart association task force on practice guidelines and the society for cardiovascular angiography and interventions. J Am Coll Cardiol.

[3] Jneid H, Anderson JL, Wright RS (2012). 2012 ACCF/AHA focused update of the guideline for the management of patients with unstable angina/non–ST-elevation myocardial infarction: a report of the American college of cardiology foundation/American heart association task force on practice guidelines. J Am Coll Cardiol.

[4] Aradi D, Komócsi A, Price MJ (2013). For the tailored antiplatelet treatment study collaboration. Efficacy and safety of intensified antiplatelet therapy on the basis of platelet reactivity testing in patients after percutaneous coronary intervention: systematic review and metaanalysis. Int J Cardiol.

[5] Ochała A, Siudak Z, Legutko J (2015). Percutaneous interventions in cardiology in Poland in the year 2014. Summary report of the association of cardiovascular interventions of the polish cardiac society AISN PTK. Postep Kardiol Inter.

[6] Bernlochner I, Steinhubl S, Braun S (2010). Association between inflammatory biomarkers and platelet aggregation in patients under chronic clopidogrel treatment. Thromb Haemost.

[7] Ahn SG, Lee SH, Yoon JH (2012). Different prognostic significance of high on-treatment platelet reactivity as assessed by the VerifyNow P2Y12 assay after coronary stenting in patients with and without acute myocardial infarction. J Am Coll Cardiol Intv.

[8] Bonello L, Camoin-Jau L, Armero S (2009). Tailored clopidogrel loading dose according to platelet reactivity monitoring to prevent acute and subacute stent thrombosis. Am J Cardiol.

[9] Hirsh J (1987). Hyperactive platelets and complications of coronary artery disease. N Engl J Med.

[10] Aradi D, Collet JP, Mair J (2015). Study group on biomarkers in cardiology of the acute cardiovascular care association of the European society of cardiology; working group on thrombosis of the European society of cardiology. Platelet function testing in acute cardiac care - is there a role for prediction or prevention of stent thrombosis and bleeding?. Thromb Haemost.

[11] Bonello L, Camoin-Jau L, Arques S (2008). Adjusted clopidogrel loading doses according to vasodilator-stimulated phosphoprotein phosphorylation index decrease rate of major adverse cardiovascular events in patients with clopidogrel resistance: a multicenter randomized prospective study. J Am Coll Cardiol.

[12] Campo G, Fileti L, de Cesare N (2010). On behalf of the 3 T/2R investigators. Long-term clinical outcome based on aspirin and clopidogrel responsiveness status after elective percutaneous coronary intervention: a 3 T/2R (tailoring treatment with tirofiban in patients showing resistance to aspirin and/or resistance to clopidogrel) trial substudy. J Am Coll Cardiol.

[13] Cuisset T, Frere C, Quilici J (2008). Glycoprotein IIb/IIIa inhibitors improve outcome after coronary stenting in clopidogrel nonresponders: a prospective, randomized study. J Am Coll Cardiol Intv.

[14] Gurbel PA, Bliden KP, Hiatt BL, O’Connor CM (2003). Clopidogrel for coronary stenting: response variability, drug resistance, and the effect of pretreatment platelet reactivity. Circulation.

[15] Campo G, Parrinello G, Ferraresi P, Lunghi B, Tebaldi M, Miccoli M (2011). Prospective evaluation of on-clopidogrel platelet reactivity over time in patients treated with percutaneous coronary intervention relationship with gene polymorphisms and clinical outcome. J Am Coll Cardiol.

[16] Gurbel PA, Tantry US (2011). An initial experiment with personalized antiplatelet therapy: the GRAVITAS trial. JAMA.

[17] Gurbel PA, Tantry US (2012). Do platelet function testing and genotyping improve outcome in patients treated with antithrombotic agents?:platelet function testing and genotyping improve outcome in patients treated with antithrombotic agents. Circulation.

[18] Tantry US, Bliden KP, Suarez TA (2010). Hypercoagulability, platelet function, inflammation and coronary artery disease acuity: results of the thrombotic risk progression (TRIP) study. Platelets.

[19] Eshtehardi P, Windecker S, Cook S (2010). Dual low response to acetylsalicylic acid and clopidogrel is associated with myonecrosis and stent thrombosis after coronary stent implantation. Am Heart J.

[20] Winter MP, Koziński M, Kubica J (2015). Personalized antiplatelet therapy with P2Y12 receptor inhibitors: benefits and pitfalls. Postep Kardiol Inter.

[21] Sibbing D, Braun S, Morath T (2009). Platelet reactivity after clopidogrel treatment assessed with point-of-care analysis and early drug-eluting stent thrombosis. J Am Coll Cardiol.

[22] Lordkipanidzé M, Pharand C, Nguyen TA (2008). Comparison of four tests to assess inhibition of platelet function by clopidogrel in stable coronary artery disease patients. Eur Heart J.

[23] Windecker S, Kolh P, Alfonso F (2014). 2014 ESC/EACTS guidelines on myocardial revascularization: the task force on myocardial revascularization of the European society of cardiology (ESC) and the European association for cardio-thoracic surgery (EACTS) developed with the special contribution of the European association of percutaneous cardiovascular interventions (EAPCI). Eur Heart J.

[24] Krishna V, Diamond GA, Kaul S (2012). Do platelet function testing and genotyping improve outcome in patients treated with antithrombotic agents?: the role of platelet reactivity and genotype testing in the prevention of atherothrombotic cardiovascular events remains unproven. Circulation.

[25] Bonello L, Tantry US, Marcucci R (2010). For the working group on high on-treatment platelet reactivity. Consensus and future directions on the definition of high on-treatment platelet reactivity to adenosine diphosphate. J Am Coll Cardiol.

[26] Uzun F, Biyik I, Akturk IF (2015). Antiplatelet resistance and the role of associated variables in stable patients treated with stenting. Postep Kardiol Inter.

[27] Mayer K, Bernlochner I, Braun S (2014). Aspirin treatment and outcomes after percutaneous coronary intervention: results of the ISAR-ASPI registry. J Am Coll Cardiol.

[28] Kirtane AJ, Parise H, Witzenbichler B (2012). Does platelet function testing add significant incremental risk stratification to unselected patients undergoing DES implantation? the ADAPT-DES study (abstr.). J Am Coll Cardiol.

[29] Hazarbasanov D, Velchev V, Finkov B (2012). Tailoring clopidogrel dose according to multiple electrode aggregometry decreases the rate of ischemic complications after percutaneous coronary intervention. J Thromb Thrombolysis.

[30] Nardin N, Verdoia M, Sartori C, et al. Diabetes mellitus, glucose control parameters and platelet reactivity in ticagrelor treated patients. Thromb Res. 2016;143:45–9. 10.1016/j.thromres.2016.04.021 27179132

[31] Thygesen K, Alpert JS, Jaffe AS (2012). Third universal definition of myocardial infarction. Eur Heart J.

[32] Cutlip DE, Windecker S, Mehran R (2007). Academic research consortium. Clinical end points in coronary stent trials: a case for standardized definitions. Circulation.

[33] Topol E, Califf R, Van de Werf F, for the GUSTO investigators (1993). An international randomized trial comparing four thrombolytic strategies for acute myocardial infarction. N Engl J Med.

[34] Hicks KA, Tcheng JE, Bozkurt B, Chaitman BR, Cutlip DE, Farb A (2014). ACC/AHA Key data elements and definitions for cardiovascular endpoint events in clinical trials: a report of the American college of cardiology/American heart association task force on clinical data standards (writing committee to develop cardiovascular endpoints data standards). J Am Coll Cardiol.

[35] Sibbing D, Schulz S, Braun S (2010). Antiplatelet effects of clopidogrel and bleeding in patients undergoing coronary stent placement. J Thromb Haemost.

[36] Tantry US, Bonello L, Aradi D (2013). Working group on on-treatment platelet reactivity. Consensus and update on the definition of on-treatment platelet reactivity to adenosine diphosphate associated with ischemia and bleeding. J Am Coll Cardiol.

[37] Breet NJ, van Werkum JW, Bouman HJ (2010). Comparison of platelet function tests in predicting clinical outcome in patients undergoing coronary stent implantation. JAMA.

[38] Komosa A, Siller-Matula JM, Lesiak M (2016). Association between high on-treatment platelet reactivity and occurrence of cerebral ischemic events in patients undergoing percutaneous coronary intervention. Thromb Res.

[39] Paniccia R, Priora R, Liotta AA, Abbate R (2015). Platelet function tests: a comparative review. Vasc Health Risk Manag.

[40] Aradi D, Kirtane A, Bonello L (2015). Bleeding and stent thrombosis on P2Y12-inhibitors: collaborative analysis on the role of platelet reactivity for risk stratification after percutaneous coronary intervention. Eur Heart J.

[41] Bonello L, Dignat-George F, Laine M (2016). Personalized antiplatelet therapy: the odyssey continues. JACC Cardiovasc Interv.

[42] Geisler T, Grass D, Bigalke B (2008). The residual platelet aggregation after deployment of intracoronary stent (PREDICT) score. J Thromb Haemost.

[43] Alexopoulos D, Xanthopoulou I, Gkizas V (2012). Randomized assessment of ticagrelor versus prasugrel antiplatelet effects in patients with ST-segment-elevation myocardial infarction. Circ Cardiovasc Interv.

[44] Dillinger JG, Saeed, A, Spagnoli, V, et al. High platelet reactivity on aspirin in patients with acute ST elevation myocardial infarction. Thromb Res. 2016;144:56–61. 10.1016/j.thromres.2016.05.002 27289074

[45] Gori AM, Cesari F, Marcucci R (2009). The balance between pro- and anti-inflammatory cytokines is associated with platelet aggregability in acute coronary syndrome patients. Atherosclerosis.

[46] Fontana P, Berdagué P, Castelli C (2010). Clinical predictors of dual aspirin and clopidogrel poor responsiveness in stable cardiovascular patients from the ADRIE study. J Thromb Haemost.

[47] Park DW, Ahn JM, Song HG (2013). Differential prognostic impact of high on-treatment platelet reactivity among patients with acute coronary syndromes versus stable coronary artery disease undergoing percutaneous coronary intervention. Am Heart J.

[48] Braun D, Knipper A, Orban M (2016). Platelet function and coagulation in patients with STEMI and peri-interventional clopidogrel plus heparin vs. Prasugrel plus bivalirudin therapy (BRAVE 4 substudy). Thromb Res.

[49] D’Ascenzo F, Bollati M, Clementi F, Castagno D, Lagerqvist B, de la Torre Hernandez JM (2013). Incidence and predictors of coronary stent thrombosis: evidence from an international collaborative meta-analysis including 30 studies, 221,066 patients, and 4276 thromboses. Int J Cardiol.

[50] Campo G, Miccoli M, Tebaldi M, Marchesini J, Fileti L, Monti M (2011). Genetic determinants of on-clopidogrel high platelet reactivity. Platelets.

[51] Stone GW, Witzenbichler B, Weisz G (2013). For the ADAPT-DES investigators. Platelet reactivity and clinical outcomes after coronary artery implantation of drug-eluting stents (ADAPT-DES): a prospective multicentre registry study. Lancet.

[52] Reny JL, Berdagué P, Poncet A (2012). For the ADRIE study group. Antiplatelet drug response status does not predict recurrent ischemic events in stable cardiovascular patients: results of the antiplatelet drug resistances and ischemic events study. Circulation.

[53] Parodi G, Marcucci R, Valenti R (2011). High residual platelet reactivity after clopidogrel loading and long-term cardiovascular events among patients with acute coronary syndromes undergoing PCI. JAMA.

[54] Mrdovic I, Čolić M, Savic L (2016). Clinical significance of laboratory-determined aspirin poor responsiveness after primary percutaneous coronary intervention. Cardiovasc Drugs Ther.

[55] Parodi G, Valenti R, Bellandi B (2013). Comparison of prasugrel and ticagrelor loading doses in ST-segment elevation myocardial infarction patients: RAPID (rapid activity of platelet inhibitor drugs) primary PCI study. J Am Coll Cardiol.

[56] Campo G, Fileti L, Valgimigli M, Tebaldi M, Cangiano E, Cavazza C (2010). Poor response to clopidogrel: current and future options for its management. J Thromb Thrombolysis.

[57] Christ G, Siller-Matula JM, Francesconi M (2014). Individualising dual antiplatelet therapy after percutaneous coronary intervention: the IDEAL-PCI registry. BMJ Open.

[58] Gurbel PA, Erlinge D, Ohman EM (2012). For the TRILOGY ACS. Platelet function substudy investigators. Platelet function during extended prasugrel and clopidogrel therapy for patients with ACS treated without revascularization: the TRILOGY ACS platelet function substudy. JAMA.

[59] Price MJ, Angiolillo DJ, Teirstein PS (2011). Platelet reactivity and cardiovascular outcomes after percutaneous coronary intervention: a time-dependent analysis of the gauging responsiveness with a VerifyNow P2Y12 assay: impact on thrombosis and safety (GRAVITAS) trial. Circulation.

[60] Price MJ, Berger PB, Teirstein PS (2011). For the GRAVITAS investigators. Standard- vs high-dose clopidogrel based on platelet function testing after percutaneous coronary intervention: the GRAVITAS randomized trial. JAMA.

[61] Trenk D, Stone GW, Gawaz M (2012). A randomized trial of prasugrel versus clopidogrel in patients with high platelet reactivity on clopidogrel after elective percutaneous coronary intervention with implantation of drug-eluting stents: results of the TRIGGER-PCI (testing platelet reactivity in patients undergoing elective stent placement on clopidogrel to guide alternative therapy with prasugrel) study. J Am Coll Cardiol.

[62] Collet JP, Cuisset T, Rangé G (2012). For the ARCTIC investigators. Bedside monitoring to adjust antiplatelet therapy for coronary stenting. N Engl J Med.

[63] Siller-Matula JM, Francesconi M, Dechant C (2013). Personalized antiplatelet treatment after percutaneous coronary intervention: the MADONNA study. Int J Cardiol.

[64] Brar SS, ten Berg J, Marcucci R (2011). Impact of platelet reactivity on clinical outcomes after percutaneous coronary intervention: a collaborative meta-analysis of individual participant data. J Am Coll Cardiol.

[65] Bonello L, Mancini J, Pansieri M (2012). Relationship between posttreatment platelet reactivity and ischemic and bleeding events at 1-year follow-up in patients receiving prasugrel. J Thromb Haemost.

[66] Mikkelsson J, Paana T, Lepantalo A, Karjalainen PP (2016). Personalized ADP-receptor inhibition strategy and outcomes following primary PCI for STEMI (PASTOR study). Int J Cardiol.

